# Long-Term Nitrogen Fertilization Elevates the Activity and Abundance of Nitrifying and Denitrifying Microbial Communities in an Upland Soil: Implications for Nitrogen Loss From Intensive Agricultural Systems

**DOI:** 10.3389/fmicb.2018.02424

**Published:** 2018-10-23

**Authors:** Fenghua Wang, Shuaimin Chen, Yuying Wang, Yuming Zhang, Chunsheng Hu, Binbin Liu

**Affiliations:** ^1^Key Laboratory of Agricultural Water Resources, Hebei Laboratory of Agricultural Water-Saving, Center for Agricultural Resources Research, Institute of Genetic and Developmental Biology, Chinese Academy of Sciences, Shijiazhuang, China; ^2^University of Chinese Academy of Sciences Beijing, China

**Keywords:** nitrogen fertilization, nitrification, denitrification, microbial community structure, high-throughput sequencing

## Abstract

The continuous use of nitrogen (N) fertilizers to increase soil fertility and crop productivity often results in unexpected environmental effects and N losses through biological processes, such as nitrification and denitrification. In this study, multidisciplinary approaches were employed to assess the effects of N fertilization in a long-term (~20 years) field experiment in which a fertilizer gradient (0, 200, 400, and 600 kg N ha^−1^ yr^−1^) was applied in a winter wheat-summer maize rotation cropping system in the North China Plain, one of the most intensive agricultural regions in China. The potential nitrification/denitrification rates, bacterial community structure, and abundances of functional microbial communities involved in key processes of the N cycle were assessed during both the summer maize (SM) and winter wheat (WW) seasons. Long-term N fertilization resulted in a decrease in soil pH and an increase in soil organic matter (OM), total N and total carbon concentrations. Potential nitrification/denitrification and the abundances of corresponding functional N cycling genes were positively correlated with the fertilization intensity. High-throughput sequencing of the 16S rRNA gene revealed that the increased fertilization intensity caused a significant decrease of bacterial diversity in SM season, while changed the microbial community composition such as increasing the *Bacteroidetes* abundance and decreasing *Acidobacteria* abundance in both SM and WW seasons. The alteration of soil properties markedly correlated with the variation in microbial structure, as soil pH and OM were the most predominant factors affecting the microbial structure in the SM and WW seasons, respectively. Furthermore, consistently with the results of functional gene quantification, functional prediction of microbial communities based on 16S rRNA sequence data also revealed that the abundances of the key nitrificaiton/denitrification groups were elevated by long-term N inputs. Taken together, our results suggested that soil microbial community shifted consistently in both SM and WW seasons toward a higher proportion of N-cycle microbes and exhibited higher N turnover activities in response to long-term elevated N fertilizer. These findings provided new insights into the molecular mechanisms responsible for N loss in intensively N fertilized agricultural ecosystems.

## Introduction

World crop production has kept growing in the past half century (Godfray et al., 2010), concomitant with an increasing nitrogen (N) fertilization intensity and a declining N use efficiency (Lu and Tian, 2017) though N use efficiency was not uniform across different countries (Zhang et al., 2015). Globally, we are applying excessive N fertilizers in agricultural systems and more than 50% of the N is lost to the environment (Luis et al., 2014), which eventually cause pollution to our ecosphere. China is now the world's largest chemical N fertilizer producer and consumer, accounting for approximately one-third of the world consumption (FAOSTAT, www.fao.org). From 1977 to 2005, N fertilizer applications increased by 271%, while grain yields increased by only 98% (Ju et al., 2009). Overuse of N fertilizer has led to serious environmental issues such as soil acidification (Guo et al., 2010), nitrate contamination in the groundwater, and greenhouse gas emissions (Liu and Zhang, 2011; Chen et al., 2014).

Recently, numerous studies have focused on the influence of the elevated N inputs on the structure of microbial communities, as a critical factor influencing terrestrial ecosystems around the world (Suding et al., 2005; Chu et al., 2007; Pan et al., 2014). It has been clearly established that the application of N fertilizer can alter the microbial taxa associated with specific components of the soil N cycle (Enwall et al., 2007; Fierer et al., 2012). However, few studies have found that N fertilization has no effect on microbial communities (Lamb et al., 2011; Carey et al., 2015). Nonetheless, the alteration of soil properties strongly correlates with the shift in the bacterial community structure. Previous studies have demonstrated that soil pH is a key environmental factor that can influence microbial community structure and can affect the distribution of bacterial phyla at a local scale (Shen et al., 2013; Jeanbille et al., 2016). In addition, the changes of soil carbon concentration and C/N ratio due to long-term N fertilization can also influence the soil microbial community structures (Marschner et al., 2003). A number of studies have showed that N input increases the soil organic carbon (SOC) over a period of long-term exposure (Christopher and Lal, 2007; Xie et al., 2017) by enhancing plant root secretion (Zhu et al., 2016) and stimulating microbes to feed on organic matter from crop residues (Gong et al., 2009). Investigation into the link between soil properties and microbial community structures is therefore essential for understanding the influence of long-term N fertilization on the function of soil microorganisms.

The oxidation of ammonia to nitrite, which is performed by ammonia-oxidizing bacteria (AOB) and archaea (AOA), is the rate-limiting step of aerobic nitrification. AOB are assumed to be the primary ammonia oxidizers in marine and terrestrial environments (Purkhold et al., 2000; Prosser and Nicol, 2008). With the discovery of AOA (Venter et al., 2004; Konneke et al., 2005; Schleper et al., 2005) and their widespread distribution in various ecosystems (Francis et al., 2005; Leininger et al., 2006; Di et al., 2010; Alves et al., 2013), the relative importance of AOA and AOB was questioned. The response of ammonia oxidizers to the input of N in soils was investigated in a number of studies, and discovered different sensitivities to plant growth stage and various forms of N in AOA and AOB (Jia and Conrad, 2009; Di et al., 2010; Wang et al., 2017).

In addition, long-term N fertilization can also strongly influence the denitrification process in soil. The increase in denitrification capacity has been reported in a number of studies, and the reasons for this phenomenon include providing a surplus of nitrate for the denitrification process (Wallenstein et al., 2006), an increase in the abundance of denitrifying bacteria (DNB) (Hallin et al., 2009) and the promotion of the organic carbon content (Simek et al., 2000). However, a cross-site study with five major Chinese upland soil samples reported no effect on the denitrification potential with successive N inputs (Qu et al., 2014). One possibility for this finding is that the factors controlling the oxic respiration rates also control the potential denitrification rates, and the key factor is likely an organic material other than the N. Similarly, no effect on the denitrification potential was reported when inorganic fertilizer was used in black soil from the Northeast China (Yin et al., 2015). Resolving this inconsistency need further in-depth investigations of the effects of N fertilization on denitrification under a broader range of soil types and N fertilization rates.

In this study, we hypothesize that long-term N input increases the abundance and activity of the functional microbial communities involved in the nitrification/denitrification processes and alters the soil microbial structures. In turn, this can potentially elevate N turnover rates, resulting in higher levels of N losses. To test this hypothesis, we chose a long-term N fertilization field experiment in which a urea fertilizer gradient (0, 200, 400, and 600 kg N ha^−1^ yr^−1^) was applied for nearly 20 years in a summer maize–winter wheat cropping system in the North China Plain. We aimed to (1) explore the changes of soil properties in the summer maize (SM) and winter wheat (WW) seasons under long-term N fertilization, (2) determine the potential rates of nitrification/denitrification and the abundances of nitrifiers and denitrifiers in the SM and WW seasons, and (3) illustrate the structural and functional differences of microbial communities under different N input intensities in the SM and WW seasons.

## Materials and methods

### Sample site and collection

The field experiment was set up in a typical fluvo-aquic soil in the Luancheng Argo-ecosystem Experimental Station, which is located in Hebei Province, China. This experiment commenced in 1998 with four treatments that used different urea application levels: N0 (no N fertilizer), N200 (200 kg N ha^−1^ yr^−1^), N400 (400 kg N ha^−1^ yr^−1^) and N600 (600 kg N ha^−1^ yr^−1^), with three replicates for each treatment (plot size 7 m × 10 m). The cropping rotation system was winter wheat–summer maize. The specific fertilization information was described previously (Qin et al., 2012a). Wheat straw and corn residues were chopped and evenly spread over each treatment area every year. Soil samples were collected in July 2015 (summer maize, SM) and January 2016 (winter wheat, WW), respectively. In each plot, five soil cores (from 0 to 15 cm) were collected along a zigzag line, and thoroughly mixed to form one composite sample. Then, soil samples were sieved (2 mm) to remove stones and roots. Each soil sample was divided into two parts: one part was kept at 4°C for chemical analyses, and the other part was stored at −80°C for molecular analyses.

### Soil physical and chemical analyses

The soil properties were measured according to the standard methods (Lu, 1999). Soil pH was measured using a pH meter (PHS-3C, Shanghai INESA) at a soil to carbon dioxide-free water ratio of 1:5. Soil water content was determined by oven drying the soil samples at 105°C for 12 h. Soil organic matter (OM) was measured using the K_2_Cr_2_O_7_ oxidation method. Total N (TN) and total carbon (TC) were determined by an Element Analyzer (Vario PYDO cube, Elementar, USA). The concentrations of nitrate (${\text{NO}}_{3}^{-}$-N) and ammonium (${\text{NH}}_{4}^{+}$-N) was measured using a spectrophotometer (SHIMADZU, UV-2450) after extracted with 2 M KCl.

### Potential nitrification activity (PNA) and potential denitrification activity (PDA)

PNA was measured using the chlorate inhibition method (Kurola et al., 2005). Briefly, 5.0 g fresh soil was weighed in a 50 ml centrifuge tube with 20 ml of phosphate buffer solution (PBS) (g/l: NaCl, 8.0; KCl, 0.2; Na_2_HPO_4_, 1.44; NaH_2_PO_4_, 0.2), 1 mM of (NH_4_)_2_SO_4_, and 10 mM of KClO_3_ [(NH_4_)_2_SO_4_ and KClO_3_ were added to inhibit nitrite oxidation], then incubated in the dark at 25°C for 24 h. Then, the nitrite was extracted with 5 ml of 2 M KCl and then measured by spectrophotometer at 540 nm with N-(1-naphthyl) ethylenediamine dihydrochloride.

PDA was measured according to the methods described by Qin et al. (2012b). Briefly, 10 g moist field soil was added to 120 ml serum flasks with 15 ml media (0.1 mM chloramphenicol, 1 mM KNO_3_, and 1 mM glucose). The flasks were sealed with butyl rubber septa and aluminum caps, and made anoxic through alternative vacuuming and filling with pure helium gas for five times. The nitrous oxide (N_2_O) and dinitrogen (N_2_) gases were then measured using an automated incubation system. The details of this system and the methods to calculate the gas production/consumption rates were described previously (Molstad et al., 2007).

### Soil DNA extraction and quantitative PCR (qPCR)

Soil total DNA was extracted using a FastDNA SPIN Kit for Soil (MP Biomedicals, USA) according to the manufacturer's instructions. The quantity and quality of the extracted DNA was measured with a NanoDrop spectrophotometer (NanoDrop ND-1000, NanoDrop Technologies, Wilmington, DE). The bacterial 16S rRNA gene was quantified using the TaqMan probe method described by Suzuki et al. (2000). Archaea 16S rRNA gene, AOB *amoA* gene, AOA *amoA* gene, *nirK, nirS*, and *nosZ* genes were amplified and quantified using a SYBR Green-based approach, and the primers and qPCR programs were described in Table S1. All qPCR reactions were performed using a Bio-Rad CFX Manager 3.1 (Bio-Rad, Hercules, CA). Each qPCR reaction was performed in a volume of 25 μl containing 12.5 μl of SYBR Premix Ex Taq (Takara Biotechnology, Dalian, China), 1 μl of each primer (10 μM), 1 μl of DNA template (approximately 10–20 ng) and 10.5 μl of dd H_2_O. Standard curves were generated using a ten-fold series dilution of the plasmids carrying the respective target genes. R^2^ values were higher than 0.997 for all calibration curves. The gene copy number was normalized to per gram of dry soil mass using the formula below:

$$\begin{array}{l} gene\;copy\;number=\frac{SQ\times D\times V}{S\times \left(1-W\right)} \end{array}$$

where SQ is the copy number of gene detected by qPCR, D is the dilution factor of DNA, V is the volume of the extracted DNA (μl), S is the weight of soil used for DNA extraction (g), and W is the soil water content.

In order to estimate the relative abundance of the functional communities within the total bacterial or archaeal communities, we calculated ratios between AOB *amoA, nirK, nirS*, and *nosZ* gene copy numbers and the bacterial 16S rRNA gene copy numbers, as well as between AOA *amoA* gene copy numbers and the archaeal 16S rRNA gene copy numbers.

### 16S rRNA gene amplification, sequencing, and data processing

The V3-V4 region of the 16S rRNA gene were amplified with primer sets 341F/785R (Yasir et al., 2015) to investigate the bacterial community diversity and structure using high-throughput sequencing technology. PCR reactions were performed in a 25 μl volume containing 12.5 μl Premix Ex Taq (Takara Biotechnology, Dalian, China), 0.5 μl forward primer (10 μM), 0.5 μl reverse primer (10 μM), 1 μl of DNA template (10–20 ng/μl) and 10.5 μl of sterilized water under the following programs: an initial denaturation at 95°C for 3 min, 23 cycles of 30 s at 95°C, 30 s at 55°C, 30 s at 72°C, and a final extension at 72°C for 5 min. The PCR products were purified using an AMPure purification system (Beckman Coulter, Danvers, MA). A subsequent eight-cycle PCR was then performed to add the Illumina sequencing adapter and dual-index barcodes for library preparation. After purification, the PCR products were sent to Shanghai Jiao Tong University (Shanghai, China) and sequenced using an Illumina Miseq system (Illumina, San Diego, USA). The high-throughput raw sequencing data were processed using the methods previously described by our group (Chen et al., 2018). Briefly, the forward and reverse reads of the PCR product were merged using FLASH (version 1.2.11) (Magoč and Salzberg, 2011) with the maximum overlap length of 170 bp. The merged sequences were filtered using the FASTX_Toolkit (http://hannonlab.cshl.edu/fastx_toolkit/). Sequences with ambiguous bases (N) and reads were shorter than 414 bp or longer than 506 bp (460 ± 10%) were discarded for further analysis. The quality of the sequences was inspected using the FastQC program (http://www.bioinformatics.babraham.ac.uk/projects/fastqc/). Detection of chimeras was performed using the UCHIME algorithm (Edgar et al., 2011). The clean data were put into the Quantitative Insights Into Microbial Ecology (QIIME) software, and then clustered into operational taxonomic units (OTUs) at the 97% similarity level using UCLUST program (Edgar, 2010), and the Greengenes database as the reference database (DeSantis et al., 2006). Functional prediction was performed using the PICRUSt software (Langille et al., 2013). Sequencing data were deposited in the European Nucleotide Archive under the accession number PRJEB26423.

### Statistical analysis

Spearman's correlation was calculated among PNA, PDA, gene abundances and soil properties using SPSS software (version 13.0), with critical *P* values corrected using Bonferroni correction. Analysis of variance (ANOVA) (Duncan) was performed to assess the significant effects of N fertilization on the soil properties, PNA, PDA, and gene abundances. Alpha diversity was assessed by calculating the Shannon, Invsimpson (the inverse of simpson index), Chao1 and PD indices. Principal Coordinate Analysis (PCoA) based on the Bray-Curtis distance was performed to visualize the bacterial community structures. Redundancy analysis (RDA), partial RDA (pRDA), and multivariate regression tree (MRT) analysis were performed in R (http://www.r-project.org) with the vegan 2.3-4 package (Oksanen et al., 2017). Permutational Multivariate Analysis of Variance (PERMANOVA) based on Bray-Curtis distance was also performed in the R package vegan with the “adonis” function. The significance of RDA analysis was tested by ANOVA based on 999 permutations. Heatmap graphs were generated using R package gpolts (Warnes et al., 2009).

## Results

### Soil properties

Long-term N fertilization resulted in pronounced effects on the soil properties during the SM and WW seasons (Table 1). In the SM season, N fertilization significantly increased the TC, TN, OM, ${\text{NO}}_{3}^{-}$-N, and ${\text{NH}}_{4}^{+}$-N concentrations (Table 1, *P* < 0.05) and significantly decreased the pH and C/N ratio (*P* < 0.05, Table 1) when compared with the N0 treatment. In the WW season, the soil properties showed trends similar to those in the SM season, except for the ${\text{NH}}_{4}^{+}$-N concentration, which decreased significantly from 0.77 ± 0.06 in N0 to 0.54 ± 0.04 in N600. In addition, soil pH and ${\text{NH}}_{4}^{+}$-N concentrations were generally higher in the SM than the WW, but the C/N ratio and the concentration of OM and ${\text{NO}}_{3}^{-}$-N were lower in the SM than in the WW (Table 1).

**Table 1:** Soil properties, PNA, and PDA in each fertilization treatment in both SM and WW seasons, respectively (Mean ± SD value).

**Items**	**SM**				**WW**			
	**N0**	**N200**	**N400**	**N600**	**N0**	**N200**	**N400**	**N600**
Soil water content	14.21 ± 1.25 b	19.18 ± 1.24 a	21.00 ± 0.38 a	20.69 ± 0.40 a	23.10 ± 2.69 b	28.74 ± 1.32 a	29.17 ± 0.13 a	26.78 ± 1.85 ab
pH	8.12 ± 0.03 a	7.95 ± 0.04 b	7.86 ± 0.03 c	7.76 ± 0.03 d	7.95 ± 0.04 a	7.59 ± 0.11 b	7.56 ± 0.11 b	7.53 ± 0.04 b
TC g kg^−1^	16.27 ± 0.38 b	19.33 ± 0.81 a	19.07 ± 0.58 a	19.40 ± 0.35 a	17.03 ± 0.70 b	20.73 ± 0.80 a	20.63 ± 0.55 a	20.80 ± 0.53 a
TN g kg^−1^	1.27 ± 0.06 b	1.60 ± 0.10 a	1.60 ± 0.01 a	1.70 ± 0.01 a	1.13 ± 0.06 b	1.57 ± 0.06 a	1.60 ± 0.10 a	1.70 ± 0.10 a
C/N	12.85 ± 0.47 a	12.09 ± 0.27 b	11.92 ± 0.36 bc	11.41 ± 0.20 c	15.04 ± 0.44 a	13.23 ± 0.22 b	12.92 ± 0.51 b	12.25 ± 0.51 b
OM g kg^−1^	18.67 ± 0.42 b	26.19 ± 0.95 a	25.50 ± 1.61 a	28.27 ± 1.04 a	23.16 ± 2.06 b	31.82 ± 0.30 a	30.55 ± 0.97 a	34.47 ± 2.70 a
${\text{NO}}_{3}^{-}$-N mg kg^−1^	12.54 ± 2.21 c	25.04 ± 2.24 b	32.27 ± 3.14 ab	33.01 ± 3.42 a	18.29 ± 5.06 c	36.91 ± 1.75 b	47.23 ± 1.94 a	51.29 ± 4.89 a
${\text{NH}}_{4}^{+}$-N mg kg^−1^	0.73 ± 0.11 b	1.66 ± 0.26 a	1.35 ± 0.21 a	1.21 ± 0.13 a	0.77 ± 0.06 a	0.48 ± 0.01 b	0.54 ± 0.05 b	0.54 ± 0.04 b
PNA	11.80 ± 0.96 c	17.82 ± 2.1 b	19.34 ± 1.29 b	19.26 ± 1.20 a	9.72 ± 0.19 b	14.40 ± 1.15 a	17.40 ± 1.53 a	17.22 ± 1.08 a
PDA	11.68 ± 1.08 b	15.73 ± 0.37 a	16.82 ± 0.97 a	19.38 ± 0.57 a	12.31 ± 0.40 b	18.63 ± 2.65 a	18.19 ± 2.12 a	21.97 ± 2.50 a

*SM, summer maize; WW, winter wheat; TC, total carbon; TN, total nitrogen; OM, soil organic matter; PNA, potential nitrification activity, unit: mg ${\text{NO}}_{2}^{-}$-N kg^−1^ dry soil d^−1^; PDA, potential denitrification activity, unit: mg N kg^−1^ dry soil d^−1^. Differing letters indicate significant differences of means in pairwise comparisons (Duncan test; P < 0.05) for each treatment in the SM and WW seasons, respectively*.

### Potential nitrification activity (PNA) and potential denitrification activity (PDA)

Long-term N inputs had a remarkably influence on the PNA and PDA during both the SM and WW seasons. In the SM season, PNA and PDA increased significantly from 11.80 ± 0.96 (N0) to 19.34 ± 1.29 (N400) and from 11.69 ± 1.08 (N0) to 19.38 ± 0.57 (N600), respectively (Table 1). While in the WW season, PNA and PDA increased significantly from 9.72 ± 0.19 (N0) to 17.40 ± 1.53 (N400) and from 12.31 ± 0.40 (N0) to 21.97 ± 2.50 (N600), respectively (Table 1). In addition, PNA was unaffected by its putative substrates ${\text{NH}}_{4}^{+}$-N, but displayed a positive correlation with ${\text{NO}}_{3}^{-}$-N (*P* < 0.05). PDA was affected by its putative substrates ${\text{NO}}_{3}^{-}$-N (*P* < 0.05), negatively correlated with pH and C/N ratio (*P* < 0.05), and positively correlated with OM and TN during both the SM and WW seasons (Tables S2, S3).

### Abundance of the bacterial and archaeal 16S rRNA genes and the functional genes

The real-time PCR assays targeting the bacterial and archaeal 16S rRNA genes yielded 2.76–6.34 × 10^10^ and 1.21–3.39 × 10^9^ copies per gram of dry soil respectively. A higher abundance was revealed for both bacteria and archaea in the N fertilized soil during both the SM and WW seasons (Figures S1A,B). The abundance values of the bacterial 16S rRNA gene in all treatments were nearly one order of magnitude higher than those of the archaeal 16S rRNA gene.

**Figure 1:**
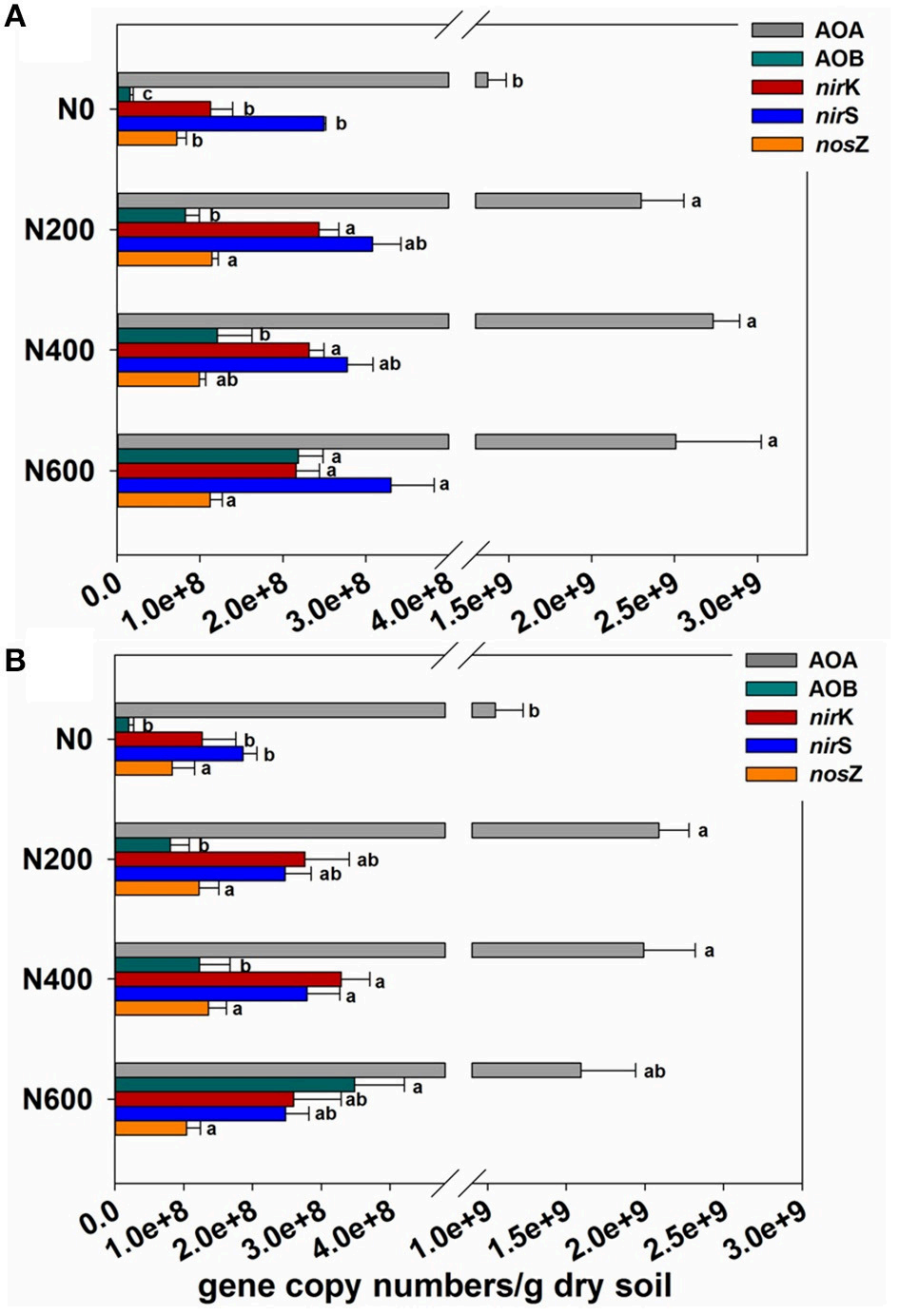
**(A,B)** The abundance of ammonia-oxidizing archaea (AOA) *amoA* gene, ammonia-oxidizing bacteria (AOB) *amoA* gene, *nirK, nirS*, and *nosZ* genes normalized to sample volume (copy numbers/g dry soil) in the SM **(A)** and WW **(B)** seasons. Error bars indicate the standard deviation of three replicates. Differing letters indicate significant differences of means of each gene in pairwise comparisons (Duncan test; *P* < 0.05) for each treatment. Means followed by the same footnote symbol(s) for each gene are not significantly different at *P* < 0.05.

The population sizes of AOA and AOB, which were determined by targeting the *amoA* gene, varied from 1.37 × 10^9^ to 2.73 × 10^9^ and 1.57 × 10^7^ to 2.18 × 10^8^ copies per gram of dry soil in SM season (Figure 1A), and from 1.05 × 10^9^ to 1.59 × 10^9^ and 1.98 × 10^7^ to 3.48 × 10^8^ copies per gram of dry soil in WW season (Figure 1B). The AOA were predominant over the AOB with a ratio of archaeal to bacterial *amoA* copy numbers during the SM and WW seasons ranging from 9.35 to 103 and 2.59 to 51.2, respectively (Figures S2A,S2B). The ratio of archaeal to bacterial *amoA* gene was also found to gradually decrease with increasing N fertilizer intensities.

For the denitrifying genes, long-term N inputs increased the abundance of the *nirK, nirS*, and *nosZ* genes in both the SM and WW seasons, except for the abundance of *nosZ* gene in the WW season. In the SM season, the abundance of the *nirK, nirS* and *nosZ* genes ranged from 1.13 × 10^8^ to 2.43 × 10^8^, 2.49 × 10^8^ to 3.30 × 10^8^, and 7.18 × 10^7^ to 1.14 × 10^8^ copies per gram of dry soil, respectively (Figure 1A). In the WW season, the abundance of the *nirK, nirS*, and *nosZ* genes ranged from 1.27 × 10^8^ to 3.29 × 10^8^, 1.86 × 10^8^ to 2.80 × 10^8^, and 8.35 × 10^7^ to 1.36 × 10^8^ copies per gram of dry soil, respectively (Figure 1B). In addition, the ratios of *nirK*/*nirS* were all below 1.0 in the SM season, whereas over 1.0 in the N fertilized soils in the WW season (Figures S2A,S2B).

According to the relative abundances of different functional communities (Figures S3A, S3B), that only bacterial *amoA* and *nir*K genes underwent significant changes during both the SM and WW seasons upon N amendments. The AOA relative abundance decreased significantly in treatments with N400 and N600 in the WW season. No significant variation in *nirS* and *nosZ* relative abundance was found in both the SM and WW seasons.

### Bacteria α-diversity, β-diversity and community composition

**Figure 2:**
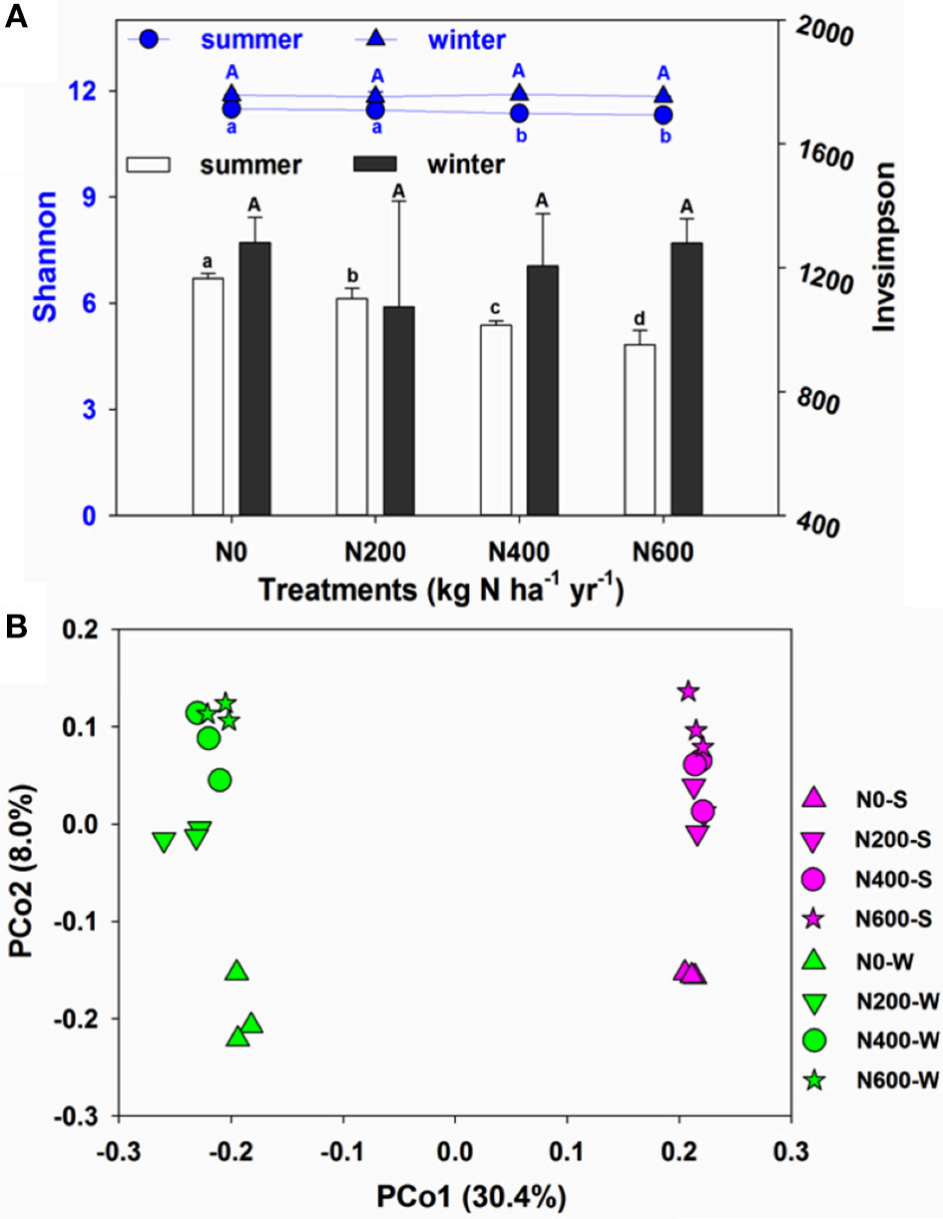
**(A)** Shannon and Invsimpson of the bacterial communities in four fertilization gradients in the SM and WW seasons. **(B)** Principal coordinate analysis (PCoA) based on the Bray-Curtis distance showing the overall distribution pattern of bacterial communities in both SM and WW seasons.

The 16S rRNA gene in each of the 24 soil samples was sequenced to elucidate the effect of long-term N fertilization on the microbial community structure. After quality control, 468,720 high quality sequences were obtained from all 24 samples (14,580–26,002 sequences per sample). These sequences were clustered into 151,917 OTUs at the 3% dissimilarity level, and the OTU numbers per sample ranged from 5444 to 7033. The bacterial richness, diversity, and evenness in the individual samples obtained from different treatments were calculated after subsampling all the samples to 14580 sequences in both the SM and WW seasons. The Shannon and Invsimpson indices showed that the N fertilization decreased the bacterial α-diversity during the SM season but not the WW season (Figure 2A). However, N fertilization had no significant influence on Chao1 and PD indices in the SM and WW seasons (Table S4). PCoA analysis revealed that the microbial community structures varied at different N levels and during different seasons (Figure 2B). According to the PERMANOVA analysis, significant differences were revealed between the bacterial community of N400, N600, and N0 treatments in the SM season, as well as between the bacterial community of N200, N600, and N0 treatments in the WW season (Table S5).

**Figure 3:**
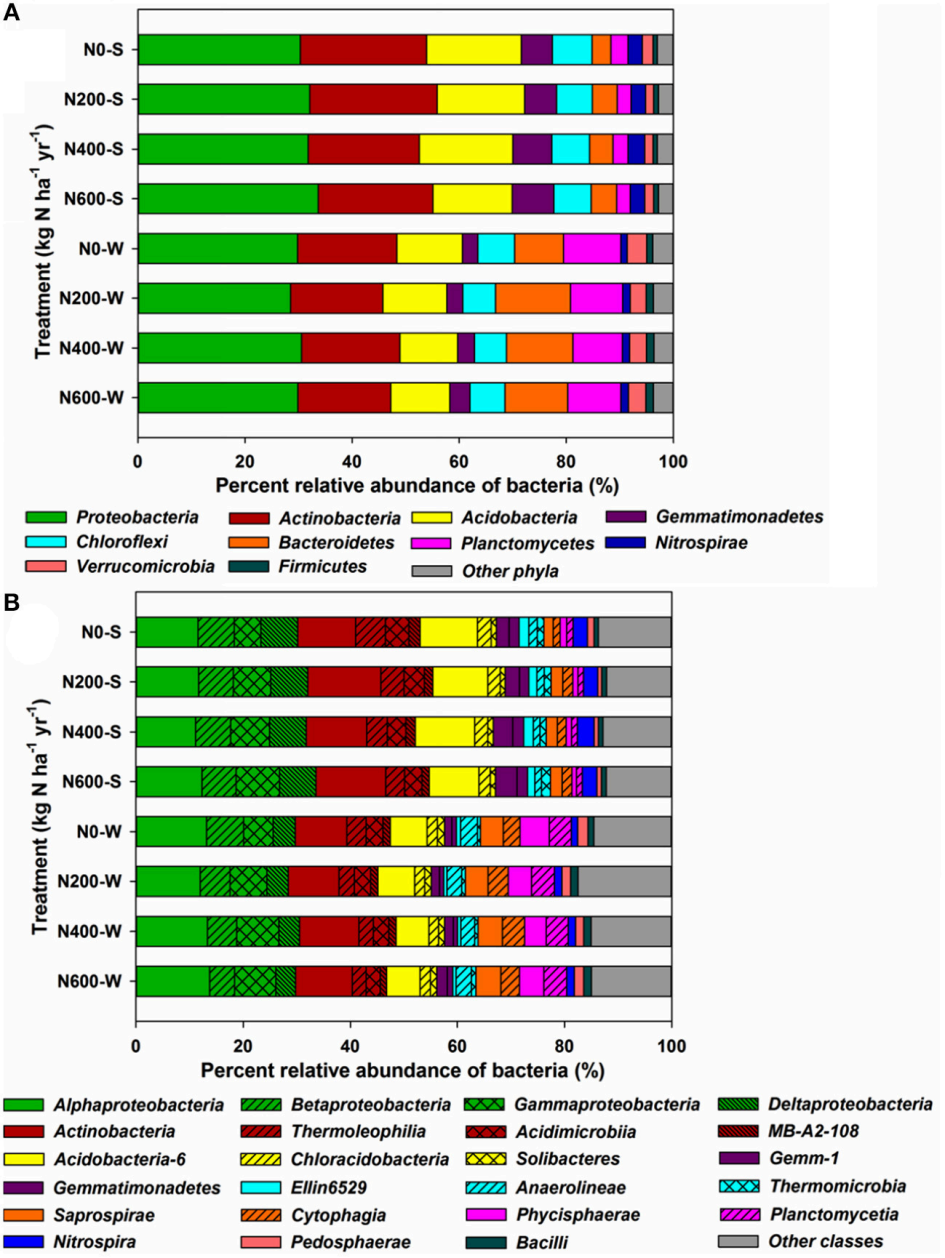
Taxonomic compositions of the dominant bacterial phyla **(A)** (relative abundance > 1%), and classes **(B)** in the SM and WW seasons. Each bar represents the average value of three replicates.

**Figure 4:**
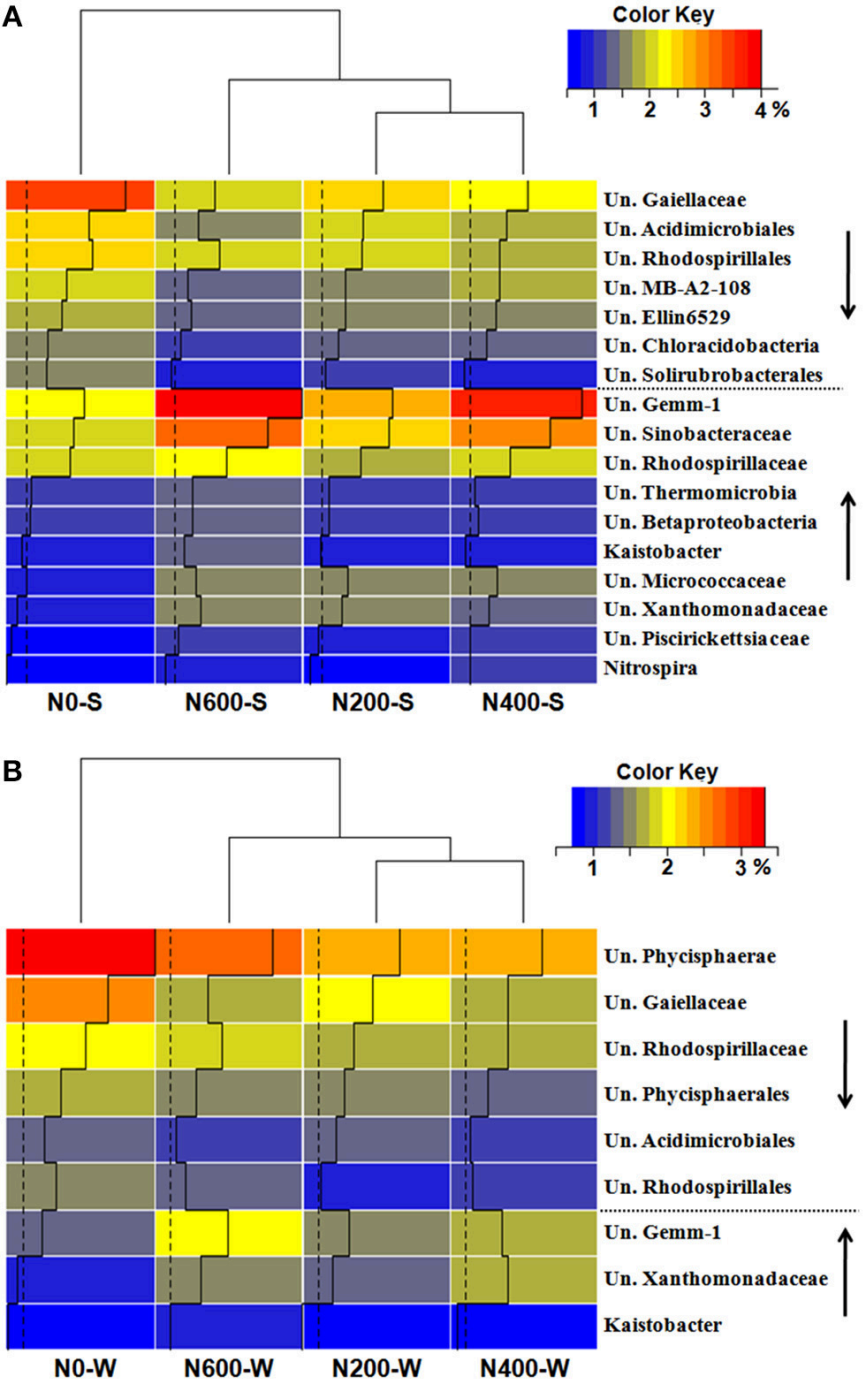
Taxonomic distributions of the dominant bacterial genus which sigfinificantly changed across fertilization intensities (relative abundance > 1%) in the SM **(A)** and WW **(B)** seasons. The downward arrow indicates a significant decrease, the upward arrow indicates a significant increase. Un, Unclassified genus.

At the phylum level, *Proteobacteria* (30.30–33.64%), *Actinobacteria* (20.72–23.75%), *Acidobacteria* (14.78–17.73%), *Gemmatimonadetes* (5.78–7.83%), and *Chloroflexi* (6.65–7.41%) were the five most abundant phyla in the SM season, accounting for more than 84% of the total bacterial relative abundance (Figure 3A). In the WW season, *Proteobacteria* (28.53–30.57%), *Actinobacteria* (17.20–18.48%), *Acidobacteria* (10.77–12.31%), *Bacteroidetes* (9.14–14.04%), and *Planctomycetes* (9.20–10.69%) were the five most abundant phyla, accounting for more than 80% of the total relative abundance (Figure 3A). Moreover, the phyla responded differently to N input levels (Table S6). For instance, *Proteobacteria* increased significantly only during the SM season. *Gemmatimonadetes* increased significantly upon N400 and N600 treatments during the SM season, but only increased significantly in N600 treatments in the WW season. *Acidobacteria* decreased significantly upon N600 treatment in the SM season and N400 treatment in the WW season (Table S6). At the class level, the differences in bacterial structures became more divergent among the different N treatments (Figure 3B). Using *Protobacteria* classes as examples, only *Gammaproteobacteria* increased significantly in the SM season, whereas the relative abundance of *Gammaproteobacteria* increased significantly upon N400 and N600 treatments and the *Betaproteobacteria* and *Deltaproteobacteria* (only in N600) classes decreased significantly during the WW season (Figure 3B, Table S7). At the genus level, 17 taxa and 9 taxa were significantly affected by N fertilizer during the SM and WW seasons, respectively (Figures 4A,B). A number of taxa were classified as unidentified genera, and in that case the higher-level taxa names preceded by an “Un.” (means unidentified genus) were shown in Figures 4A,B.

### Correlation among the abundance of functional and phylogenetic genes with PNA, PDA, and soil properties

No significant correlation was found between soil properties and the abundance of bacterial and archaeal 16S rRNA genes (*P* > 0.05, after *P* values were Bonferroni-corrected) during both the SM and WW seasons (Tables S2,S3). PNA had a significantly positive correlation with the abundance of AOB *amoA* gene during the SM (*r* = 0.76, *P* < 0.05) and WW (*r* = 0.74, *P* < 0.05) seasons, whereas PDA had a significant correlation with the abundance of *nirS* gene (*r* = 0.86, *P* < 0.01) specifically during the WW season (Tables S2,S3). For the nitrifying genes, archaeal *amoA* gene was only positively correlated with TC (*r* = 0.78, *P* < 0.05), whereas bacterial *amoA* gene was negatively correlated with pH (*r* = −0.90, *P* < 0.01) and C/N ratio (*r* = −0.89, *P* < 0.01) and positively correlated with OM, ${\text{NO}}_{3}^{-}$-N, and TC (*r* = 0.90, 0.87, 0.88, respectively, *P* < 0.01) during the SM season (Table S2). During the WW season, only bacterial *amoA* gene had a significant negative correlation with C/N ratio (*r* = −0.87, *P* < 0.01) and was positively correlated with ${\text{NO}}_{3}^{-}$-N, TC, and TN (*r* = 0.93, 0.82, 0.92, respectively) (Table S3). For the denitrifying genes, there was no significant correlation between denitrification genes with soil properties during the SM and WW seasons, with the exception of the *nirS* gene, which had significant correlation with pH (*r* = −0.85, *P* < 0.01), C/N ratio (*r* = −0.81, *P* < 0.05), TC (*r* = 0.78, *P* < 0.05) and TN (*r* = 0.86, *P* < 0.01) (Table S3) in the WW season. In general, only bacterial *amoA* gene and *nirS* gene were significantly related to soil properties in comparison to the other functional genes.

### Correlation between bacterial community structure and soil properties

**Figure 5:**
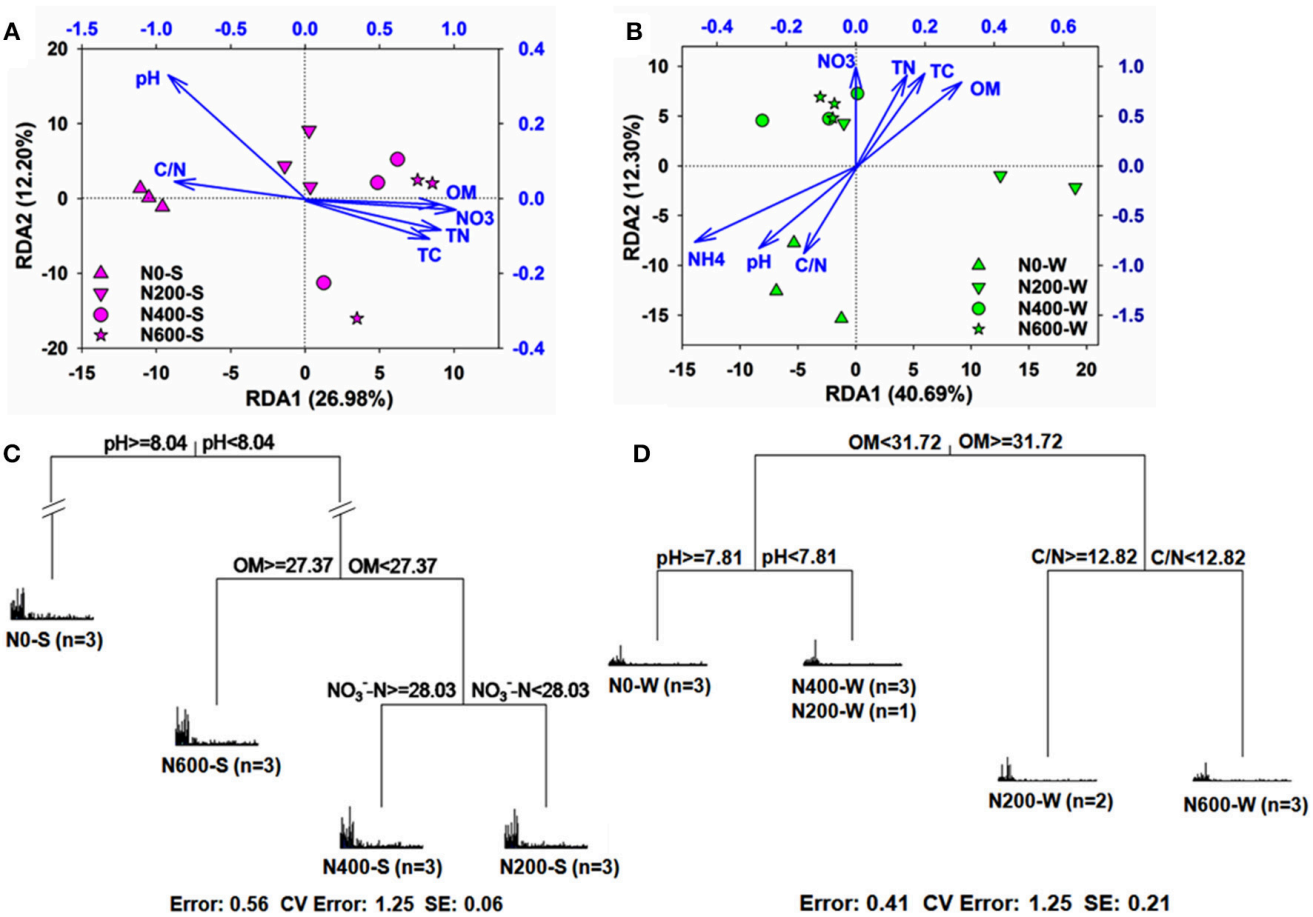
**(A,B)** Redundancy analysis (RDA) plots depict the correlation between bacterial communities and soil properties in the SM (**A**, *P* = 0.001) and WW (**B**, *P* = 0.039) seasons; **(C,D)** Multivariate regression tree (MRT) analysis of bacterial communities under different N treatments in the SM **(C)** and WW **(D)** seasons. Specific treatment and the number of samples included in the analysis are shown under bar plots. OM, soil organic matter; NO_3_/${\text{NO}}_{3}^{-}$-N, nitrate concentration; NH4, ammonium; TC, total carbon; TN, total N; C/N: C/N ratio. Different shapes of symbols represent different treatments.

RDA and pRDA analysis were performed to evaluate the correlations between microbial communities and soil properties. Soil properties explained 66.40 and 76.69% of the total variation in the soil bacterial community structure during the SM and WW seasons, as determined by RDA, and their first two axis explained 26.98 and 12.20%, 40.69 and 12.30%, respectively (Figures 5A,B). Six soil properties from the SM season (pH, ${\text{NO}}_{3}^{-}$-N, TC, TN, OM, and C/N ratio) and seven soil properties from the WW season (OM, TC, TN, pH, ${\text{NO}}_{3}^{-}$-N, ${\text{NH}}_{4}^{+}$-N, and C/N ratio) were identified as important factors that contributed to the variation in the bacterial community according to the results of pRDA (Table S8). In addition, the MRT analysis showed that the microbial communities could be distinguished by pH, OM, and ${\text{NO}}_{3}^{-}$-N during the SM season, and OM, pH, and C/N ratio during the WW season. Soil pH and OM were the main factors that contributed to the variation of microbial structure during the SM and WW seasons, respectively (Figures 5C,D). Furthermore, the relative abundance of certain phyla was more dependent than others on the soil properties in the SM and WW seasons (Table S9). During the SM season for example, the soil pH had a significant negative correlation with *Proteobacteria, Gemmatimonadetes*, and *Bacteroidetes* (*r* = −0.81, −0.77, −0.69, respectively, *P* < 0.01), while OM had a positive correlation with *Proteobacteria* (*r* = 0.83, *P* < 0.01) and *Gemmatimonadetes* (*r* = 0.68, *P* < 0.05), and a negative correlation with *Acidobacteria* (*r* = −0.62, *P* < 0.05) and *Planctomycetes* (*r* = −0.78, *P* < 0.01). During the WW season, the soil pH had a negative correlation with *Bacteroidetes* (*r* = −0.64, *P* < 0.05) and a positive correlation with *Planctomycetes* (*r* = 0.71, *P* < 0.01), whereas OM only significantly correlated with *Verrucomicrobia* (*r* = −0.71, *P* < 0.01).

## Discussion

Globally the terrestrial ecosystems are receiving increasing amount of N input from anthropogenic activities, largely via the N fertilizers (Vitousek et al., 2009). The increased N fertilization has led to improved agro-environmental performances in some developed countries, while produced higher environmental losses in other areas, especially developing countries (Luis et al., 2014). Chinese farmlands are treated with unnecessarily high levels of N fertilizers (Ju et al., 2009), and recent studies have proposed solutions to this issue, including increasing the farm size (Wu et al., 2018). Understanding of how elevated N availability affects soil microbial community structures, abundance of the functional groups, and potential N turnover activities is essential for developing new techniques to improve the fertilization efficiency and mitigate environmental damage. Although similar research has been conducted with the black soil from the Northeast China (Zhou et al., 2017), the dramatic heterogeneity in soil properties across the country (Shi et al., 2004) necessitates site- or soil-type-specific studies.

In this study, the long-term N fertilization altered soil nutrient concentrations and pH (Table 1). Interestingly, we found that ${\text{NH}}_{4}^{+}$-N and soil pH was generally higher during the SM season than the WW season. As the temperature is lower during the WW season, it was assumed that ${\text{NH}}_{4}^{+}$-N consumption rates was higher than gross N mineralization, resulting in a lower concentration and availability of soil ${\text{NH}}_{4}^{+}$-N than during the SM season (Hoyle et al., 2006; Dong et al., 2012). Moreover, when compared with N0 treatment, the soil pH decreased by 0.4 and 0.5 units with fertilization treatment in SM and WW seasons, respectively (Table 1). The lower soil pH in the WW season compared with SM season is likely a result of the mineralization of higher amount of corn residues in the WW season, as various acidic compounds are generated during the process of cellulose degradation (Kato et al., 2005). Soil acidification is becoming a major problem for global terrestrial ecosystems, and it has been recently recognized as a serious issue in Chinese agricultural systems that stems from the incomplete cycling of N species and the input of acidifying N fertilizers in the soil (Guo et al., 2010). Furthermore, nitrification, particularly the ammonia oxidation improved by N inputs, can also decrease the soil pH (Pernes-Debuyser and Tessier, 2004).

Long-term N fertilization increased the PNA during the SM and WW seasons when compared to that of unfertilized soil, this result was similar with previous findings (Chu et al., 2007; Shen et al., 2008). The increased nitrification likely contributes to a higher potential of N loss and greenhouse gas (N_2_O) emission (Shen et al., 2012). In addition, there was a significant correlation between the PNA and the abundance of bacterial *amoA* gene (*P* < 0.05), whereas no significant correlation between the PNA and the abundance of archaeal *amoA* gene, although AOA had higher abundance than AOB. In line with this result, Shen et al. also found that PNA significantly correlated with AOB abundance, but not with that of AOA (Shen et al., 2008). The decline in the ratio of the archaeal to bacterial *amoA* gene copy numbers with increasing N input levels during both SM and WW seasons (Figures 1A,B) could likely be attributed to improved soil fertility under long-term N fertilization, since fertilization likely favors the growth of AOB rather than AOA, as AOA are better adapted to low-fertility or oligotrophic environments (Erguder et al., 2009; Di et al., 2010). More sensitive response to urea amendment of AOB than AOA observed in this study was possibly due to that certain AOB can produce urease and are therefore capable of utilizing urea for chemolithotrophic growth (Koper et al., 2004).

The PDA was also elevated by long-term N fertilization during both the SM and WW seasons when compared with unfertilized soil. However no significant associations were determined between PDA and denitrification genes except the *nirS* gene in the WW. Although the *nir* and *nos* genes have been used as indicators for the potential of denitrification (Bru et al., 2011; Philippot et al., 2011) and their ratio for denitrification derived N_2_O flux (Simek and Cooper, 2002), recent studies have revealed that potential denitrification are strongly regulated at the post-transcriptional levels, and this has been proved both in a pure culture of microorganisms (Bergaust et al., 2010) and in soils (Liu et al., 2010). In addition, the functional genes, like atypical *nosZ* gene, which were not included in this study, could also influence the PDA. In another ongoing project where the same soils used in this study were analyzed with metagenomic sequencing technology, we have found that the abundance of atypical *nosZ* was even higher than typical *nosZ* (Paper in prep). It is therefore not surprising to obtain a poor correlation between PDA and the abundance of individual denitrification gene, which indicating that doing this type of statistical analysis may be of trivial importance.

Functional prediction using the PICRUSt software indicated that the key nitrification/denitrification functional groups represented by ammonia monooxygenase, nitrite reductase, and nitrous oxide reductase (Figure S6) were increased upon N fertilization compared to unfertilized soil even though the differences among the fertilization intensities were not always significant. In addition, statistic analysis showed significant correlations between the abundance of *nosZ* gene and the relative abundance of nitrous oxide reductase (K00376) (*r* = 0.50) (Figure S7B), as well as between the PDA and the relative abundance of nitrite reductase (K00368) (r = 0.73) (Figure S7C) and nitrous-oxide reductase (K00376) (*r* = 0.49) (Figure S7D). No significant correlation was found between the abundance of (*nirK* + *nirS*) genes and the relative abundance of nitrite reductase (K00368) (*P* > 0.05) (Figure S7A). The main families of ammonia oxidizers, *Nitrosomonadaceae* and *Nitrospiraceae*, were inspected and found enriched upon long-term N fertilization (Figure S4). Both of these two families significantly correlated with the abundance of bacterial *amoA* gene (*r* = 0.80, 0.47, respectively) (Figures S5A,B) and also clearly correlated with PNA though the correlation with *Nitrosomonadaceae* was not significant (*P* = 0.102) (Figures S5C,D). These analyses indicated that the results achieved from the 16S rRNA gene sequencing supported the results from quantification of functional genes and also our hypothesis.

Long-term N fertilization decreased the bacterial diversity significantly during the SM season, but not during the WW season. A number of studies have found that N fertilization reduced the bacterial diversity of soil (Campbell et al., 2010; Zhou et al., 2017) while no significant changes in bacterial diversity among different N fertilizer levels was also observed (Fierer et al., 2012), suggesting that the influence of N amendments on bacterial diversity were inconsistent and likely site-dependent. Soil pH and OM were identified as the most predominant factors in explaining the differentiation of the microbial community structure across the N fertilization intensities during the SM and WW seasons, respectively. Soil pH has also been reported in previous studies to be a crucial environmental factor determining the community structure of soil bacteria (Shen et al., 2013; Sun et al., 2015). Rather than dramatic switches between dominant and rare taxa, moderate shifts in the relative abundances of the microorganisms were observed between the SM and WW seasons, this season variation of microbial responses to N fertilization may also be due to different crop species in the two seasons since plant can dramatically influence the surrounding soil and its microflora (Hartmann et al., 2009).

## Conclusions

This study investigated the effects of long-term N fertilization on soil PNA and PDA, the structure and function of soil microbial communities in an upland agricultural ecosystem during both the SM and WW seasons. Long-term N fertilization has elevated the potential nitrification and denitrification activities and also brought about significant changes in a plethora of soil properties. The abundance of nitrifier/denitrifier which is responsible for N turnover in soil was increased by long-term N fertilization, as consistently revealed by both quantification of the functional genes and functional prediction with the 16S rRNA gene sequence data. Soil pH and OM were identified as the most predominant factors involved in differentiating the microbial structure in the SM and WW seasons, respectively. The results of this study provide novel insights into the responses of soil microbiota to long-term N input and molecular mechanisms responsible for N loss in intensively N fertilized agricultural ecosystems.

## Author contributions

FW and SC collected soil samples, performed laboratorial measurement, and data analysis. YW, YZ, and CH designed and managed the experimental field. FW and BL wrote this paper. All authors read and approved the final version of the manuscript.

### Conflict of interest statement

The authors declare that the research was conducted in the absence of any commercial or financial relationships that could be construed as a potential conflict of interest.
