# Supplementary material for: Long-Term Nitrogen Fertilization Elevates the Activity and Abundance of Nitrifying and Denitrifying Microbial Communities in an Upland Soil: Implications for Nitrogen Loss From Intensive Agricultural Systems
Source: Front Microbiol. 2018 Oct 23;9:2424. doi: 10.3389/fmicb.2018.02424 (PMC6206047; doi:10.3389/fmicb.2018.02424)
Supplement: Supplementary file 1 [file Data_Sheet_1.PDF]

Long-term nitrogen fertilization elevates the activity and abundance of nitrifying and denitrifying microbial communities in an upland soil: implications for nitrogen loss from intensive agricultural systems

Fenghua Wang[a], Shuaimin Chen[a,b], Yuying Wang[a], Yuming Zhang[a], Chunsheng Hu[a], Binbin Liu[a*]

[a] Key Laboratory of Agricultural Water Resources, Hebei Laboratory of Agricultural Water-Saving, Center for Agricultural Resources Research, Institute of Genetic and Developmental Biology, Chinese Academy of Sciences, Shijiazhuang 050021, China

[b] University of Chinese Academy of Sciences, Beijing 100039, China

*Corresponding author:
Binbin Liu
E-mail address: binbinliu@sjziam.ac.cn
Tel: +86 311 85817713; fax: +86 311 85815093

Article Type: Original Research
Number of Figures: 7
Number of Tables: 9

Table S1 Primers, probes and PCR conditions used for qPCR.

Target group	Primer and probe	Sequence (5′-3′)	Length of amplicon (bp)	PCR conditions	Reference
Bacteria	Primer 1369F	CGGTGAATACGTTCYCGG	150	95°C 3min, followed by 40 cycles of 10 s at 95°C, 60 s at 55°C, plate read at 55°C	(Suzuki et al., 2000)
	Primer 1492R	GGWTACCTTGTTACGACTT			
	Probe 1389F	CTTGTACACACCGCCCGTC			
Archaea	A364aF	CGGGGYGCASCAGGCGCGAA	550	95°C 3min, followed by 40 cycles of 30 s at 95°C, 40 s at 59°C, 40 s at 72°C, plate read at 83°C	(Kemnitz et al., 2005)
	A934bR	GTGCTCCCCCGCCAATTCCT			
AOA	Arch-amoAF	STAATGGTCTGGCTTAGACG	635	95°C 3min, followed by 40 cycles of 40 s at 95°C, 40 s at 53°C, 40 s at 72°C, plate read at 83°C	(Francis et al., 2005)
	Arch-amoAR	GCGGCCATCCATCTGTATGT			
AOB	amoA-1F	GGGGTTTCTACTGGTGGT	491	95°C 3min, followed by 40 cycles of 40 s at 95°C, 40 s at 60°C, 40 s at 72°C, plate read at 83°C	(Rotthauwe et al., 1997)
	amoA-2R	CCCCTCKGSAAAGCCTTCTTC			
nirK	nirK-FlaCu	ATCATGGTSCTGCCGCG	472	95°C 3min, followed by 40 cycles of 30 s at 95°C, 40 s at 57°C, 40 s at 72°C, plate read at 83°C	
	nirK-R3Cu	GCCTCGATCAGRTTGTGGTT			
nirS	nirS-cd3aF	GTSAACGTSAAGGARACSGG	425	95°C 3min, followed by 40 cycles of 30 s at 95°C, 40 s at 56.8°C, 40 s at 72°C, plate read at 83°C	(Throback et al., 2004)
	nirS-R3cd	GASTTCGGRTGSGTCTTGA			
nosZ	nosZ-1F	CGYTGTTCMTCGACAGCCAG	453	95°C 3min, followed by 40 cycles of 30 s at 95°C, 40 s at 56.8°C, 40 s at 72°C, plate read at 83°C	
	nosZ-1622R	CGSACCTTSTTGCCSTYGCG			

Francis, C.A., Roberts, K.J., Beman, J.M., Santoro, A.E., Oakley, B.B. (2005). Ubiquity and diversity of ammonia-oxidizing archaea in water columns and sediments of the ocean. *Proc. Natl. Acad. Sci. U.S.A.* 102, 14683-14688.

Kemnitz, D., Kolb, S., Conrad, R. (2005). Phenotypic characterization of Rice Cluster III archaea without prior isolation by applying quantitative polymerase chain reaction to an enrichment culture. *Environ. Microbiol.* 7, 553-565.

Rotthauwe, J.H., Witzel, K.P., Liesack, W. (1997). The ammoina monnoxygenase structural gene *amoA* as a functional marker: molecular fine-scale analysis of natural ammonia-oxidizing populations. *Appl. Environ. Microbiol.* 63, 4704-4712.

Suzuki, M.T., Taylor, L.T., DeLong, E.F. (2000). Quantitative analysis of small-subunit rRNA genes in mixed microbial populations via 5 '-nuclease assays. *Appl. Environ. Microbiol.* 66, 4605-4614.

Throback, I.N., Enwall, K., Jarvis, A., Hallin, S. (2004). Reassessing PCR primers targeting *nirS*, *nirK* and *nosZ* genes for community surveys of denitrifying bacteria with DGGE. *FEMS Microbiol. Ecol.* 49, 401-417.

Table S2 Spearman's correlation among PNA, PDA, and gene abundances with soil properties in the SM season.

	r value									*P* value, Bonferroni-corrected								
	pH	OM	NO_3^--N	NH_4^+-N	TC	TN	C/N	PNA	PDA	pH	OM	NO_3^--N	NH_4^+-N	TC	TN	C/N	PNA	PDA
PNA	-0.64	0.55	0.87**	0.47	0.32	0.50	-0.61			0.355	0.875	0.003	1.000	1.000	0.969	0.315		
PDA	-0.87**	0.85**	0.89**	0.41	0.53	0.75*	-0.76*	0.83**		0.003	0.006	0.001	1.000	0.821	0.048	0.034	0.006	
Bact	-0.57	0.51	0.39	0.44	0.69	0.70	-0.60	0.51	0.49	0.761	1.000	1.000	1.000	0.133	0.105	0.352	0.719	0.744
Arch	-0.66	0.69	0.75	0.29	0.60	0.65	-0.66	0.71	0.76*	0.298	0.195	0.067	1.000	0.432	0.211	0.184	0.074	0.028
AOA	-0.71	0.52	0.63	0.49	0.78*	0.63	-0.52	0.62	0.64	0.143	1.000	0.368	1.000	0.033	0.275	0.730	0.265	0.183
AOB	-0.90**	0.82*	0.87**	0.35	0.60	0.88**	-0.89**	0.76*	0.90**	0.001	0.016	0.003	1.000	0.449	0.001	0.001	0.036	0.001
nirK	-0.48	0.41	0.40	0.71	0.65	0.53	-0.40	0.59	0.43	1.000	1.000	1.000	0.112	0.254	0.731	1.000	0.353	1.000
nirS	-0.54	0.62	0.48	0.16	0.50	0.70	-0.67	0.55	0.61	1.000	0.429	1.000	1.000	1.000	0.111	0.154	0.533	0.251
nosZ	-0.07	0.24	0.45	0.39	0.02	0.05	-0.21	0.63	0.38	1.000	1.000	1.000	1.000	1.000	1.000	1.000	0.227	1.000

Correlation is significant at the 0.05 level (*), 0.01 level (**) (2-tailed).

Table S3 Spearman's correlation among PNA, PDA, and gene abundances with soil properties in the WW season.

	r value									*P* value, Bonferroni-corrected								
	pH	OM	NO_3^--N	NH_4^+-N	TC	TN	C/N	PNA	PDA	pH	OM	NO_3^--N	NH_4^+-N	TC	TN	C/N	PNA	PDA
PNA	-0.58	0.29	0.75**	-0.29	0.43	0.56	-0.66			0.736	1.000	0.067	1.000	1.000	0.592	0.182		
PDA	-0.85**	0.84**	0.75	-0.58	0.75	0.92**	-0.92**	0.48		0.007	0.009	0.067	0.597	0.059	0.000	0.000	0.897	
Bacteria	-0.68	0.52	0.44	-0.71	0.60	0.61	-0.56	0.25	0.60	0.215	1.000	1.000	0.123	0.452	0.344	0.527	1.000	0.270
Archaea	-0.65	0.65	0.63	-0.57	0.59	0.69	-0.67	0.42	0.68	0.312	0.308	0.368	0.618	0.468	0.136	0.151	1.000	0.107
AOA	-0.73	0.61	0.41	-0.70	0.52	0.59	-0.62	0.41	0.62	0.102	0.501	1.000	0.143	0.927	0.432	0.299	1.000	0.232
AOB	-0.71	0.72	0.93**	-0.44	0.82*	0.92**	-0.87**	0.74*	0.85**	0.151	0.115	0.000	1.000	0.011	0.000	0.002	0.042	0.004
nirK	-0.73	0.55	0.62	-0.56	0.62	0.72	-0.71	0.54	0.72	0.102	0.875	0.431	0.730	0.333	0.084	0.092	0.567	0.058
nirS	-0.85**	0.71	0.63	-0.55	0.78*	0.86**	-0.81*	0.48	0.86**	0.008	0.126	0.349	0.772	0.031	0.003	0.013	0.915	0.002
nosZ	-0.54	0.34	0.41	-0.43	0.46	0.53	-0.50	0.18	0.50	1.000	1.000	1.000	1.000	1.000	0.789	0.905	1.000	0.704

Correlation is significant at the 0.05 level (*), 0.01 level (**) (2-tailed).

Table S4 The value of chao1, PD_whole_tree, and OTU numbers (OTUs) in both SM and WW seasons. (Average ± SD value)

Items	SM				WW			
	N0	N200	N400	N600	N0	N200	N400	N600
chao1	13819.5±486.3 a	13937.7±830.4 a	13841.8±1943.2 a	13445.1±673.0 a	19168.4±932.3 A	19181.5±502.3 A	20260.7±611.2 A	19606.3±912.3 A
PD	250.8±2.4 a	250.7±3.0 a	245.9±15.5 a	242.0±3.6 a	320.7±2.0 A	319.6±4.9 A	324.4±2.4 A	318.6±3.9 A
OTUs	5696±88 a	5677±45 a	5499±294 a	5444±97 a	6902±66 A	6872±172 A	7033±36 A	6867±77 A

Note: Differing letters indicate significant differences of means in pairwise comparisons (Duncan test; $P < 0.05$) for each treatment. SM: lowercase letters; WW: uppercase letters.

Table S5 Permutational Multivariate Analysis of Variance (PERMANOVA) examined the difference of microbial communities among four different N fertilization levels in both SM and WW seasons.

**SM**	N0	N200	N400
N200	0.099		
N400	0.001**	0.107	
N600	0.001**	0.094	0.099

**WW**	N0	N200	N400
N200	0.001**		
N400	0.103	0.001**	
N600	0.001**	0.090	0.018

R values were calculated based on 999 permutations
Significance level: $P < 0.05$, *; $P < 0.01$, **.

Table S6 Phyla that significantly increased or decreased under different N fertilization levels in both SM and WW seasons.

	SM				
phylum	N0-S	N200-S	N400-S	N600-S	
Proteobacteria	30.30±0.99 c	32.10±0.81 b	31.81±0.37 b	33.64±0.35 a	**increase**
Acidobacteria	17.73±0.13 a	16.34±1.67 ab	17.45±0.58 a	14.78±1.59 b	**decrease**
Gemmatimonadetes	5.78±0.18 b	6.00±0.36 b	7.27±0.39 a	7.83±0.78 a	**increase**
Chloroflexi	7.41±0.31 a	6.65±0.05 b	7.08±0.40 ab	6.91±0.19 ab	**decrease**
Bacteroidetes	3.49±0.26 b	4.64±0.54 a	4.36±0.67 ab	4.74±0.37 a	**increase**
Planctomycetes	3.23±0.25 a	2.62±0.07 b	2.80±0.24ab	2.57±0.16 b	**decrease**
Verrucomicrobia	2.02±0.08 a	1.57±0.13 b	1.58±0.21 b	1.72±0.11 ab	**decrease**

phylum	WW				
	N0-W	N200-W	N400-W	N600-W	
Acidobacteria	13.31±0.48 a	11.93±0.17 ab	10.77±0.58 b	11.01±1.18 ab	**decrease**
Gemmatimonadetes	2.89±0.19 b	3.01±0.03 b	3.19±0.11 b	3.80±0.39 a	**increase**
Bacteroidetes	9.14±1.05 b	14.04±2.65 a	12.42±0.8 ab	11.79±1.11 ab	**increase**
Planctomycetes	10.69±0.63 a	9.69±0.15 ab	9.20±0.47 b	9.84±0.18 ab	**decrease**
Verrucomicrobia	3.71±0.11 a	3.00±0.13 b	3.16±0.15 ab	3.32±0.29 ab	**decrease**

Differing letters indicate significant differences of means in pairwise comparisons (Duncan test; $P < 0.05$) for each treatment.

Table S7 Classes that significantly increased or decreased under different N fertilization levels in both SM and WW seasons..

Phylum	Class	SM				
		N0-S	N200-S	N400-S	N600-S	
Proteobacteria	*Gammaproteobacteria*	4.96±0.46 b	7.04±0.57 a	7.27±0.14 a	8.13±0.38 a	**increase**
Actinobacteria	*Thermoleophilia*	5.57±0.19 a	4.27±0.17 b	3.82±0.07 bc	3.55±0.33 c	**decrease**
Actinobacteria	*Acidimicrobiia*	4.38±0.15 a	3.79±0.12 b	3.48±0.03 c	3.22±0.12 d	**decrease**
Actinobacteria	*MB-A2-108*	2.07±0.18 a	1.59±0.08 b	1.77±0.34 ab	1.34±0.21 b	**decrease**
Acidobacteria	*Chloracidobacteria*	2.57±0.19 a	2.31±0.2 ab	2.42±0.28 ab	2.12±0.22 b	**decrease**
Gemmatimonadetes	*Gemm-1*	2.36±0.05 b	2.66±0.19 b	3.64±0.40 a	4.01±0.61 a	**increase**
Chloroflexi	*Anaerolineae*	1.58±0.10 a	1.33±0.08 b	1.25±0.03 b	1.25±0.11 b	**decrease**
Chloroflexi	*Thermomicrobia*	1.21±0.02 b	1.33±0.09 b	1.17±0.10 b	1.66±0.12 a	**increase**
Planctomycetes	*Phycisphaerae*	1.23±0.18 a	1.00±0.06 b	1.00±0.10 b	0.86±0.08 b	**decrease**
Planctomycetes	*Planctomycetia*	1.19±0.05 a	0.99±0.03 b	1.08±0.11 ab	1.07±0.07 ab	**decrease**
Verrucomicrobia	*Pedosphaerae*	1.22±0.03 a	0.77±0.09 b	0.83±0.18 b	0.89±0.11 b	**decrease**

Phylum	Class	WW				
		N0-W	N200-W	N400-W	N600-W	
Proteobacteria	*Alphaproteobacteria*	13.19±1.04 ab	12.01±0.19 b	13.37±0.24 ab	13.73±0.84 a	**increase**
Proteobacteria	*Betaproteobacteria*	6.89±1.01 a	5.54±0.15 b	5.41±0.31 b	4.66±0.37 b	**decrease**
Proteobacteria	*Gammaproteobacteria*	5.54±0.80 b	6.86±0.39 ab	7.90±0.69 a	7.73±0.89 a	**increase**
Proteobacteria	*Deltaproteobacteria*	4.11±0.20 a	4.06±0.20 a	3.84±0.18 ab	3.70±0.11 b	**decrease**
Actinobacteria	*Thermoleophilia*	3.60±0.38 a	2.87±0.09 b	2.73±0.19 b	2.56±0.12 b	**decrease**
Actinobacteria	*Acidimicrobiia*	3.14±0.10 a	3.04±0.19 a	2.87±0.19 ab	2.69±0.17 b	**decrease**
Acidobacteria	*Solibacteres*	1.35±0.04 a	1.22±0.02 ab	1.12±0.05 b	1.20±0.12 ab	**decrease**
Gemmatimonadetes	*Gemm-1*	1.33±0.13 c	1.54±0.05 bc	1.64±0.02 b	2.00±0.30 a	**increase**
Chloroflexi	*Ellin6529*	0.78±0.04 a	0.60±0.05 b	0.63±0.04 b	0.66±0.02 ab	**decrease**

Chloroflexi	*Anaerolineae*	3.13±0.28 a	2.76±0.07 ab	2.59±0.15 b	2.84±0.33 ab	**decrease**
Chloroflexi	*Thermomicrobia*	0.58±0.06 b	0.66±0.01 b	0.63±0.07 b	0.85±0.06 a	**increase**
Planctomycetes	*Phycisphaerae*	5.54±0.60 a	4.37±0.23 b	4.07±0.42 b	4.54±0.23 ab	**decrease**

Differing letters indicate significant differences of means in pairwise comparisons (Duncan test; $P < 0.05$) for each treatment.

Table S8 Explained variance by partial RDA analysis in both SM and WW seasons.

Factors	Explained variance	
	**SM**	**WW**
pH	11.64%***	5.85%**
OM	6.24%**	20.43%***
TC	7.88%**	7.13%**
TN	7.65%**	9.77%***
NO_3^--N	9.97%***	7.57%**
NH_4^+-N	NS	5.61%**
C/N	6.98%**	8.33%**

Significance level: P < 0.05, *; P < 0.01, **; P < 0.001, ***.

Table S9 Spearman's correlation analysis among soil properties and main phyla (>1%).

**SM**	pH	OM	NO_3^--N	NH_4^+-N	TC	TN	C/N
Proteobacteria	-0.81**	0.83**	0.76**	0.27	0.45	0.76**	-0.83**
Actinobacteria	0.46	-0.22	-0.22	-0.28	-0.38	-0.27	0.25
Acidobacteria	0.44	-0.62*	-0.55	0.06	-0.10	-0.48	0.62*
Gemmatimonadetes	-0.77**	0.68*	0.80**	0.44	0.54	0.56	-0.47
Chloroflexi	0.15	-0.25	-0.10	-0.40	-0.20	-0.35	0.40
Bacteroidetes	-0.69*	0.52	0.57	0.36	0.47	0.76**	-0.85**
Planctomycetes	0.51	-0.78**	-0.78**	-0.54	-0.50	-0.61*	0.53
Nitrospirae	-0.18	-0.07	-0.02	0.28	0.32	0.04	0.11
Verrucomicrobia	0.35	-0.54	-0.63*	-0.72**	-0.53	-0.40	0.28
Firmicutes	-0.42	0.22	0.23	0.10	0.12	0.45	-0.63*

**WW**	pH	OM	NO_3^--N	NH_4^+-N	TC	TN	C/N
Proteobacteria	-0.09	-0.39	0.27	0.34	-0.09	0.06	-0.11
Actinobacteria	0.28	-0.45	-0.10	0.19	0.04	-0.19	0.41
Acidobacteria	0.44	-0.17	-0.72**	0.30	-0.29	-0.44	0.52
Gemmatimonadetes	-0.38	0.42	0.77**	-0.06	0.37	0.60*	-0.69*
Chloroflexi	0.34	-0.08	-0.37	0.61*	-0.12	-0.12	0.20
Bacteroidetes	-0.64*	0.49	0.35	-0.85**	0.33	0.36	-0.44
Planctomycetes	0.71**	-0.48	-0.51	0.58*	-0.42	-0.52	0.58*
Nitrospirae	-0.52	0.55	0.20	-0.28	0.45	0.45	-0.45
Verrucomicrobia	0.48	-0.71**	-0.32	0.59*	-0.42	-0.39	0.35
Firmicutes	-0.17	0.28	0.42	-0.26	0.41	0.25	-0.14

Correlation is significant at the 0.05 level (*), 0.01 level (**) or at the 0.001 level (***) or not significant at 0.05 level (NS) (2-tailed)

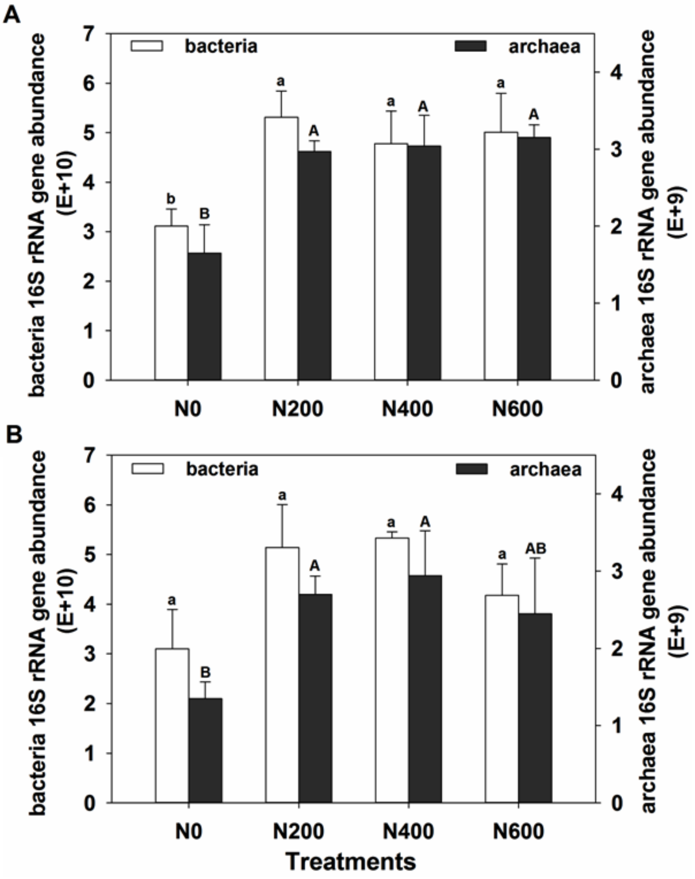

Figure S1. The abundance of bacteria 16S rRNA gene and archaea 16S rRNA gene in SM (A) and WW (B) seasons (copies per g dry soil). Differing letters indicate significant differences of means in pairwise comparisons (Duncan test; $P < 0.05$) for each treatment. bacteria: lowercase letters; archaea: uppercase letters. Error bars indicate the standard deviation of three replicates.

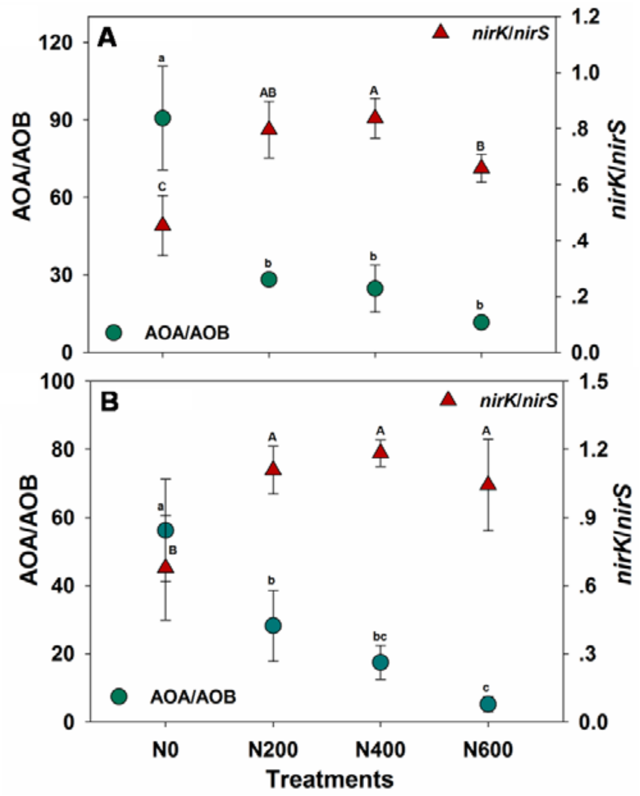

Figure S2. The ratio of AOA *amoA* gene/AOB *amoA* gene, and the ratio of *nirK*/*nirS* gene in SM (A) and WW (B) seasons. Differing letters indicate significant differences of means in pairwise comparisons (Duncan test; $P < 0.05$) for each treatment. AOA/AOB: lowercase letters; *nirK*/*nirS*: uppercase letters. Error bars indicate the standard deviation of three replicates.

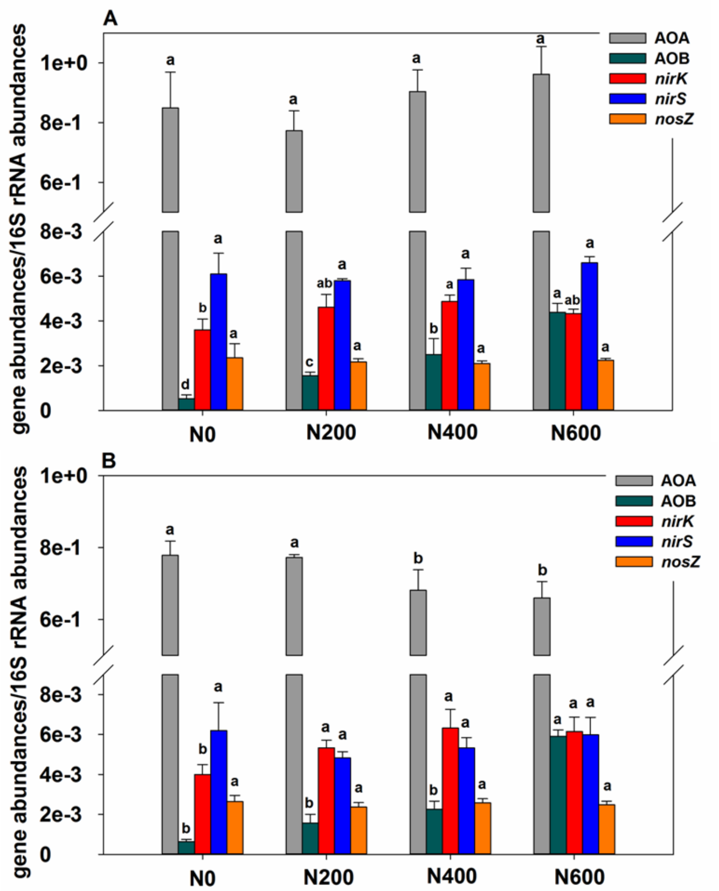

Figure S3. A and B: the relative abundance of ammonia-oxidizing archaeal (AOA) *amoA* gene copies normalized to archaea 16S rRNA gene copies, and ammonia-oxidizing bacterial (AOB) *amoA* gene, *nirK*, *nirS*, and *nosZ* gene copies normalized to bacteria 16S rRNA gene copies in the SM (A) and WW (B) seasons.

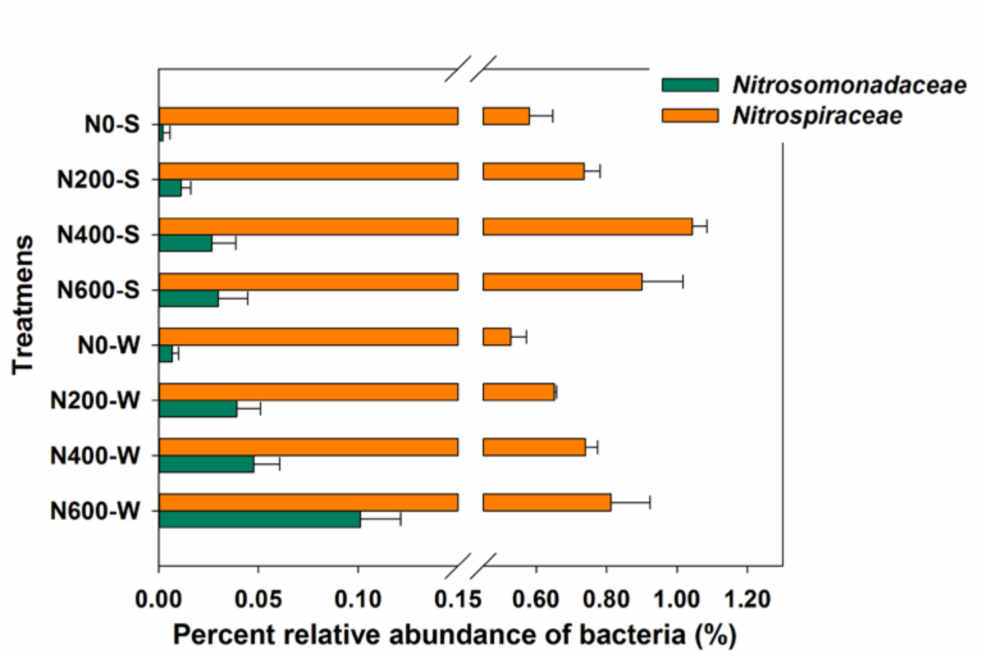

Figure S4. Percent relative abundance of the family *Nitrosomonadaceae* and *Nitrospiraceae* in SM and WW seasons. Each bar represents the average value of three replicates. S: SM season; W: WW season.

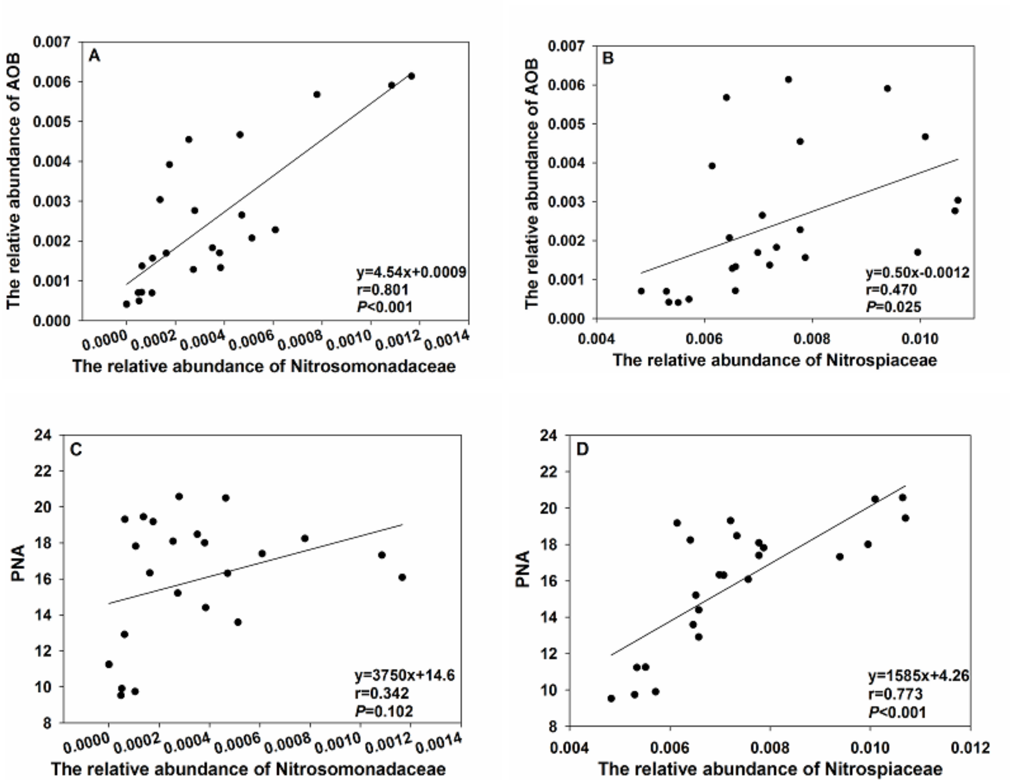

Figure S5. A and B: Correlation between the abundance of AOB (bacterial *amoA* gene) and the relative abundance of *Nitrosomonadaceae* and *Nitrospiraceae*, respectively. C and D: Correlation between the abundance of PNA and the relative abundance of *Nitrosomonadaceae* and *Nitrospiraceae*, respectively.

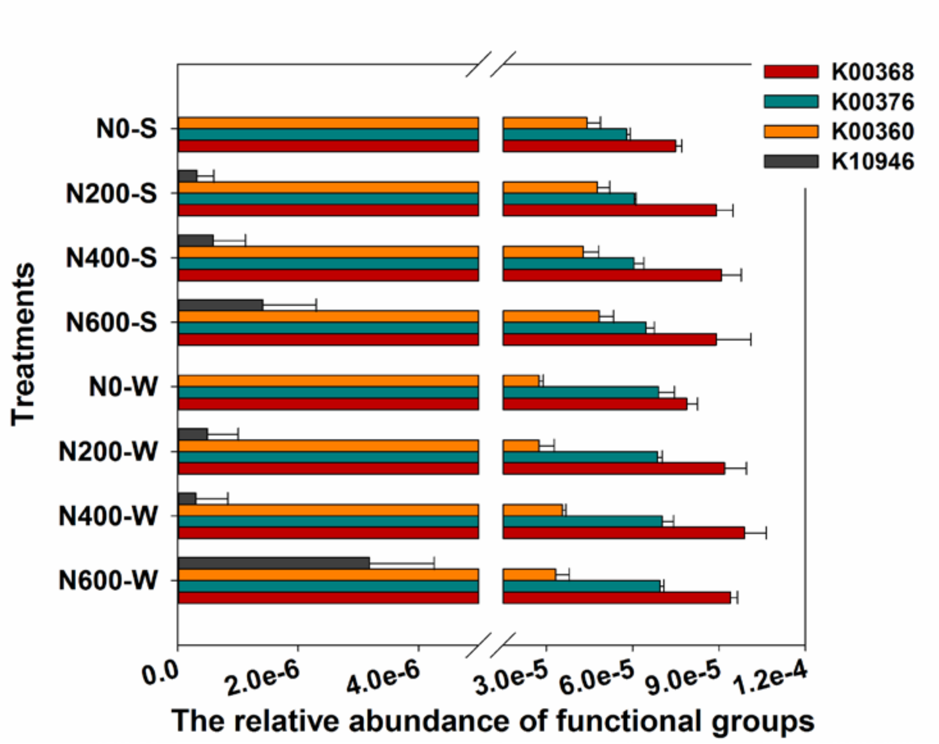

Figure S6. The relative abundance of nitrogen functional groups predicted using PICRUSt with 16S rRNA gene sequences. K00368: nitrite reductase (NO-forming) (EC:1.7.2.1); K00376: nitrous-oxide reductase (EC:1.7.99.6); K00360: nitrate reductase (NADH) (EC:1.7.1.1); K10946: ammonia monooxygenase subunit C (EC:1.13.12.)

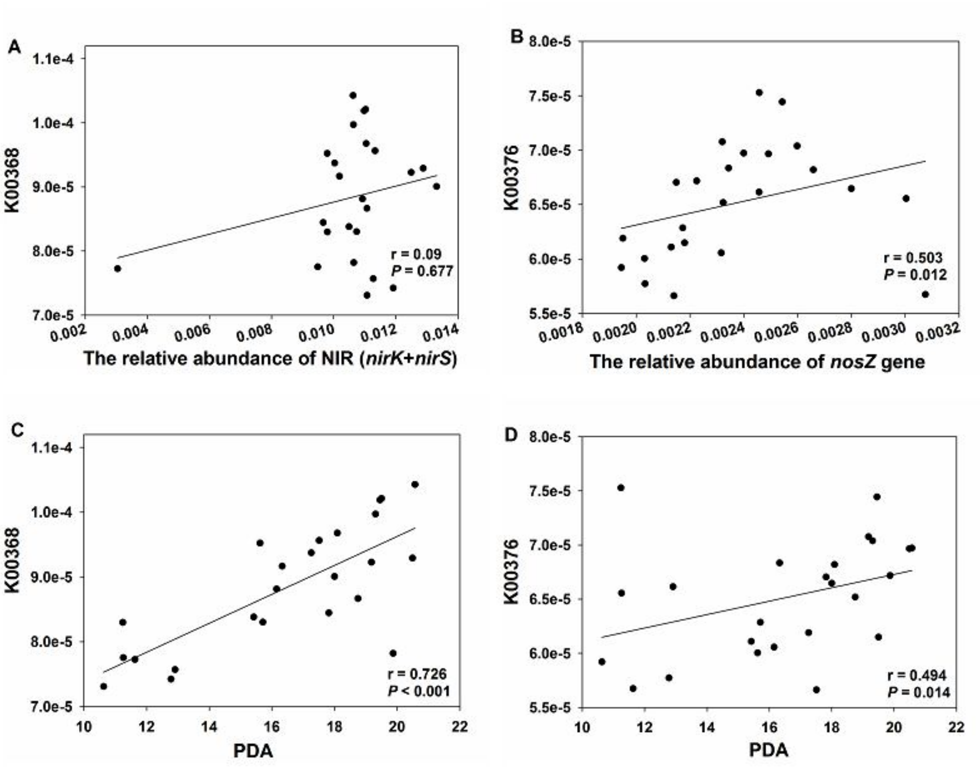

Figure S7. A: Correlation between the abundance of (*nirK*+*nirS*) genes and the relative abundance of K00368; B: Correlation between the abundance of *nosZ* gene and the relative abundance of K00376; C and D: Correlation between PNA and the relative abundance of K00368 and K00376, respectively. K00368: nitrite reductase (NO-forming) (EC:1.7.2.1); K00376: nitrous-oxide reductase (EC:1.7.99.6);
